# Thermophoretic analysis of ligand-specific conformational states of the inhibitory glycine receptor embedded in copolymer nanodiscs

**DOI:** 10.1038/s41598-020-73157-2

**Published:** 2020-10-06

**Authors:** Max Bernhard, Bodo Laube

**Affiliations:** 1 Department of Biology, Neurophysiology and Neurosensory Systems, Technical University of Darmstadt, Schnittspahnstrasse 3, 64287 Darmstadt, Germany; 2 Centre for Synthetic Biology, Technical University of Darmstadt, 64283 Darmstadt, Germany

**Keywords:** Biochemistry, Biophysics, Structural biology, Neuroscience, Ion channels in the nervous system

## Abstract

The glycine receptor (GlyR), a member of the pentameric ligand-gated ion channel family (pLGIC), displays remarkable variations in the affinity and efficacy of the full agonist glycine and the partial agonist taurine depending on the cell system used. Despite detailed insights in the GlyR three-dimensional structure and activation mechanism, little is known about conformational rearrangements induced by these agonists. Here, we characterized the conformational states of the α1 GlyR upon binding of glycine and taurine by microscale thermophoresis expressed in HEK293 cells and *Xenopus* oocytes after solubilization in amphipathic styrene-maleic acid copolymer nanodiscs. Our results show that glycine and taurine induce different conformational transitions of the GlyR upon ligand binding. In contrast, the variability of agonist affinity is not mediated by an altered conformational change. Thus, our data shed light on specific agonist induced conformational features and mechanisms of pLGIC upon ligand binding determining receptor activation in native environments.

## Introduction

The superfamily of pentameric ligand-gated ion channels (pLGIC) mediates excitatory and inhibitory synaptic neurotransmission in the central nervous system^1^ and is the target for many therapeutic agents^2^. The knowledge of the underlying mechanisms determining apparent affinity and efficacy of small ligands acting on these receptors is fundamental for the understanding of their physiological and pharmacological properties under developmental, normal and pathological conditions of the brain. One of the most pressing questions regarding ligand-specific activity concerns the underlying mechanisms by which partial and full agonists possess variable half-maximal effective concentration (EC_50_) values and amplitude responses under different cellular conditions. Despite detailed insights into their three-dimensional structures and general activation mechanisms, an accurate knowledge of the underlying structural confinements determining the affinity and efficacy of agonists at pLGICs is still lacking^1,3^. The inhibitory glycine receptor (GlyR), a member of the pLGICs, is an outstanding example displaying exceptional variations in the affinity and efficacy of its agonists^4^. GlyRs mediate, together with GABA receptors, the majority of fast inhibitory neurotransmission in the central nervous system and are implicated in neuronal diseases like hypereplexia^4,5^ and forms of epilepsy^6^. GlyRs assemble as homopentamers of α-subunits (α1–4) or heteropentamers, containing both α- and β-subunits^7^ and are composed of an extracellular orthosteric ligand binding site (ECD), a transmembrane spanning ion-channel (TMD) and an intracellular domain (ICD). GlyR activation upon agonist binding induces a contraction within the ECD that is transmitted to the TMD leading to the opening of the chloride selective intrinsic channel pore^8^. Remarkably, the efficacy to open the ion channel depends on the agonist used, i.e. glycine acts as a full agonist inducing maximal current responses with opening times of 95–98% seen in single-channel recordings^9^, whereas taurine acts as a partial agonist producing a decreased maximal response relative to the effect produced by glycine that is reflected by lower open-channel probabilities^10^. However, both the efficacy and affinity of glycine and taurine also strongly depend on the expression system used^4,11^ and can vary up to an order of magnitude when heterologously expressed in HEK293 cells^12,13^ and *X. laevis* oocytes^14,15^. Further*,* the relative efficacy of taurine of heterologous expressed α1 GlyR is directly correlated with its apparent affinity^15^ indicating a dependency of taurine-affinity and -efficacy in heterologous expression systems. Interestingly, these results cannot be dismissed as expression system dependent phenomena because the taurine efficacy and affinity similarly varies in magnitude in different brain regions^16–18^, indicating that mechanisms regulating the efficacy of the partial agonist taurine in the brain are related to differences also seen in heterologous expression systems. However, despite several available crystal structures of glycine receptors^8,19^ and extended kinetic models based on single-channel recordings^10,20^, so far little is known about the specific conformational rearrangements of the GlyR during receptor activation induced by full and partial agonists and even less which conformations can be adopted during activation in different cell systems. Thus, structural aspects of the relative affinity and efficacy of ligands to open the intrinsic ion channel within specific native environments, a core question in receptor pharmacology, may be ideally investigated at the GlyR by analyzing ligand-specific conformational states under native conditions.

Here we report the combination of detergent-free isolation of the heterologous expressed α1 GlyR in HEK293 cells and *X. laevis* oocytes by amphipathic styrene-maleic acid (SMA)-copolymers with microscale thermophoresis (MST) as a new approach to study structural impacts of full and partial agonists for the GlyR conformation. Upon successful solubilization of α1 GlyR from HEK293 cells and *X. laevis* oocytes in nanodiscs we can show by MST that both the affinity and efficacy of the full agonist glycine to induce a conformational change was identical in both expression systems although the respective electrophysiological EC_50_ values differed by an order of magnitude. In contrast, the partial agonist taurine stabilized a distinct conformational state that can be clearly distinguished from the conformational state adopted after glycine binding. Thus, we provide experimental evidence for the underlying mechanism of partial agonism at the GlyR and that variations in EC_50_ values observed in different expression systems are likely mediated by an impaired ability of the receptor to open the channel once the agonist has bound.

## Materials and methods

### GlyR constructs

For heterologous expression in HEK293 cells and *X. laevis* oocytes, human his tagged α1-His GlyR^21^ and N-terminal GFP fused α1-GFP GlyR was cloned into pCDNA3.1(+) vector by using NotI and NheI (Thermo Fisher Scientific, Waltham, MA, USA). All constructs were confirmed by sequencing (Seqlab, Göttingen, Germany).

### Heterologous expression of α1 GlyR in HEK293 cells

HEK293 cells were cultured in minimum essential medium (MEM) supplemented with 10% (v/v) FCS, 2 mM l-glutamine and streptomycin (100 µg/ml) at 37 °C and 5% CO_2_. For transfection, 14–20 × 10^6^ cells were diluted in electroporation buffer 1 M (5 mM KCl, 15 mM MgCl_2_, 120 mM Na_2_HPO_4_/NaH_2_PO_4_ pH 7.2, 50 mM mannitol) at a final concentration of 1 × 10^6^ cells per 100 µl and 500 ng per 100 µl of the respective plasmid was added. Electroporation was performed using the Amaxa Nucleofector II S system (Lonza, Basel, Switzerland). After transfection, cells were reseeded in 75 cm^2^ flasks with MEM and incubated for 48 h.

### Ethical approval

All methods involving animals were carried out in accordance with the guidelines and regulations of the local animal care and use committee. Methods were approved by the Technical University of Darmstadt (II25.3-19c20/15, RP Darmstadt, Germany).

### Heterologous expression of α1-GFP GlyR in *X. laevis* oocytes

cRNA was synthesized using the AmpliCap-Max T7 High Yield Message Maker Kit (Cellscript, Madison, WI, USA). Therefore GFP-GlyR *α1* in pCDNA3.1(+) was linearized with NotI. Oozytes were surgically taken from female *X. laevis* after anesthesia with 0.1% Tricaine in water. For SMA-copolymer solubilization, 300–400 oocytes were injected with 50 ng in a volume of 50.6 nl of cRNA. After injection the oocytes were incubated in ND-96 solution (96 mM NaCl, 2 mM KCl, 1 mM CaCl_2_, 1 mM MgCl_2_, 5 mM HEPES, pH 7.4) at 18 °C for 1–2 days.

### SMA-copolymer solubilization of α1 GlyR

For SMA copolymer solubilization of the heterogeneous expressed α1 GlyR in HEK293 cells, cells were washed with PBS, subsequently scraped off and resuspended in 2 ml PBS. Cells were washed and resuspended in 150 mM NaCl, 50 mM Tris/HCl pH 8.0 and lysed by sonification. Membranes are separated by ultracentrifugation at 100,000*g* for 1.5 h and 4 °C. For α1 GlyR expressed in oocytes, cells were resuspended in 20 mM Tris–HCl, pH 8.0 and mechanically homogenized by pipetting. To remove cell debris, cells were centrifuged at 1000×*g* for 15 min at 4 °C. Membranes were separated by ultracentrifugation at 100,000×*g* for 1 h at 4 °C. To remove additional yolk proteins^22^, pellet was washed with 1 M NaCl, 20 mM Tris–HCl, pH 8.0, followed by an additional ultracentrifugation step as written above. The membrane pellet was resuspended in SMA-solubilization buffer (150 mM NaCl, 10% glycerol, 50 mM Tris/HCl pH 8.0, SIGMAFAST Protease Inhibitor Cocktail (Sigma-Aldrich, St. Louis, MO, USA) to a concentration of 80 mg/ml. A freshly prepared 4% (w/v) SMA copolymer solution (Lipodisq Styrene:Maleic Anhydride Copolymer 3:1, Pre-hydrolyzed, Sigma-Aldrich) in SMA-solubilization buffer was slowly dropped under stirring to the membrane suspension in a 1:1 ratio and was solubilized for 1 h at room temperature. To remove all non-solubilized cell fragments, the suspension was centrifuged at 100,000*g* for 45 min and 4 °C.

### Ni-NTA purification and size exclusion chromatography

The α1-His GlyR SMA copolymer nanodiscs containing supernatant was incubated with pre-equilibrated 0.2 ml HisPur Ni-NTA spin columns (Thermo Fisher Scientific) overnight at 4 °C under gentle rotation. Spin columns were washed three times with SMA-solubilization buffer supplemented with 25 mM imidazole and α1-His GlyR SMALPs were eluted in SMA-solubilization buffer supplemented with 400 mM imidazole. For separation of the previously purified α1 GlyR nanodiscs from other soluble proteins and for further analysis, pooled elution fractions were loaded on a Superdex 200 increase 10/300 GL (GE Healthcare, Chicago, IL, USA) connected to an Äkta pure (GE Healthcare) and buffer was exchanged to 50 mM Tris/HCl pH 7.4 and 150 mM NaCl. Estimation of the molecular weight (MW) was based on a calibration curve by a linear fit of proteins of known MW (Aldolase, Ovalbumin, Conalbumin, Cyanocobalbumin, Thyroglobulin) versus the partition coefficient k_av_ (k_av_ = V_el_ − V_0_/V_t_ − V_o_, where V_el_ is the elution volume of the protein, V_t_ is the total column volume and V_0_ is the void volume). The hydrodynamic radii (R_s_, Stokes radius) where calculated, according to the Stokes–Einstein relation^23^, by a linear fit of the Stokes radii of known proteins versus the square root of the negative decadic logarithm of the k_av_.

### SDS-PAGE and western blot

SDS-PAGE and western blot analysis was performed as described elsewhere^24^. In brief, proteins were separated using a 10% sodium dodecyl sulfate polyacrylamide gel electrophoresis (SDS-PAGE). For SDS-PAGE analysis, the gel was stained with Pierce Silver Stain Kit (Thermo Fisher Scientific). For western blot analysis, separated proteins were transferred to a PVDF membrane (Bio-Rad, Feldkirchen, Germany) and the membrane was blocked for 1 h in TBS-T supplemented with 5% skim milk. Afterwards, the membrane was incubated with 1:500 primary eGFP Polyclonal antibody (CAB4211, Thermo Fisher Scientific) in TBS-T containing 1% skim milk over night at 4 °C. The membrane was washed 3 times for 10 min with TBS-T and incubated with the goat anti-rabbit IgG-HRP (sc-2054, Santa Cruz Biotechnology, Dallas, TX, USA) in TBS-T containing 1% skim milk for 1 h at room temperature. The membrane was washed 3 times for 10 min in TBS-T and protein bands were visualized by adding Pierce Western Blotting Substrate (Thermo Fisher Scientific) and detected with a CCD camera.

### Microscale thermophoresis

Microscale thermophoresis (MST) analysis was performed using a NanoTemper Monolith NT.115 instrument (NanoTemper Technologies, Munich, Germany). Therefore, purified α1-His GlyR nanodiscs or total solubilized cell membranes with α1-GFP GlyR were diluted to a concentration of 400 nM in PBS. α1-His GlyR was fluorescence-labeled using the Monolith NT His-Tag Labeling Kit RED-tris-NTA (NanoTemper Technologies). Labeled GlyR α1 SMALPs were added in a 1:1 ratio to a 1:2 dilution series with a final concentration of 3 mM down to 0.73 µM for glycine or 12.5 mM down to 6 µM for taurine, as well as 0 µM for each ligand as an internal control and loaded into standard capillaries (Monolith NT.115 Capillaries, NanoTemper Technologies). Thermophoresis was measured at 21 °C for 15 or 20 s with 40% LED power and 60% infrared laser power. For MST experiments n = 3–4 independent technical measurements were collected from N = 2–3 independent oocyte or HEK293 cell batches.

### Electrophysiological recordings

1–2 days after injection of α1 GlyR cRNA in *X. laevis* oocytes, whole-cell currents were recorded by two-electrode voltage-clamp using an Axoclamp 900A amplifier and a Digidata 1550A digitizer. Data were sampled at 5 kHz after low-pass filtering at 200 Hz and recorded with Clampex 10.7 (Molecular Devices, San Jose, USA). For recordings, oocytes were clamped at − 70 mV in external Ringer solution (115 mM NaCl, 1 mM KCl, 0.9 mM CaCl_2_, 10 mM HEPES, pH 7.4). For HEK293, whole-cell recordings of GlyR α1 transfected cells were carried out as described in Laube et al. 2000^25^. In brief, whole cell currents were recorded 2 days after transfection using an EPC-9 amplifier (HEKA, Ludwigshafen, Germany) and data were sampled at 20 Hz. Patch pipettes contained 120 mM CsCl, 20 mM TEA-Cl, 1 mM CaCl_2_, 2 mM MgCl_2_, 11 mM EGTA, 10 mM HEPES, pH 7.2. Membrane potential was clamped at − 70 mV and cells were perfused with external solution (137 mM NaCl, 5.4 KCl, 1.8 mM CaCl_2_, 1 mM MgCl, 5 mM HEPES, pH 7.4). Increasing glycine concentrations were applied using a microcapillary application system (DAD-12, Adams and List, Westbury, NY, USA).

### Data and statistical analysis

For electrophysiological dose–response analysis, normalized current responses were plotted against the agonist concentration and fitted with a sigmoidal Hill equation $I/I_{max} = 100 \times c^{n} /(c^{n} + aEC_{50}^{n} )$ in GraphPad Prism 8 (GraphPad Software Inc., La Jolla, USA), where *I/I*_*max*_ is the normalized current, *c* the concentration, *n* the Hill coefficient and *aEC*_*50*_ the agonist concentration resulting in a half-maximal response. For thermophoretic binding experiments, the relative thermophoretic fluorescence signal F_norm_ was calculated as ratio of the initial fluorescence (F_cold_ = 1 s) and fluorescence after thermodiffusion (F_hot_ = 15 s) using the following equation in MO.Affinity Analysis software (NanoTemper Technologies): $F_{norm} = F_{hot} /F_{cold} = 1 + (\partial F/\partial T - S_{T} )\Delta T$, where ∂F/∂T is the fluorescence change due to the fluorophore’s temperature dependence and S_T_ the Soret coefficient. For thermophoretic dose–response analysis, the normalized relative fluorescences were plotted against the agonist concentration and fitted with the sigmoidal Hill equation $F_{norm} /F_{norm,max} = 100/(1 + (cEC_{50} /c)^{n} )$, where *F*_*norm*_*/F*_*norm,max*_ is the normalized relative fluorescence, *cEC*_*50*_ the agonist concentration resulting in a half-maximal response and *n* the Hill coefficient. All values are given in mean ± SEM, unless indicated otherwise. Statistical significance was determined using a Student’s two-tailed, unpaired two-side *t* test with *p < 0.05, **p < 0.01, and ***p < 0.001 levels. Equality of variances was confirmed using a *F* test.

## Results

### Detergent-free purification of homomeric α1 GlyR in SMA-copolymer nanodiscs

**Figure 1:**
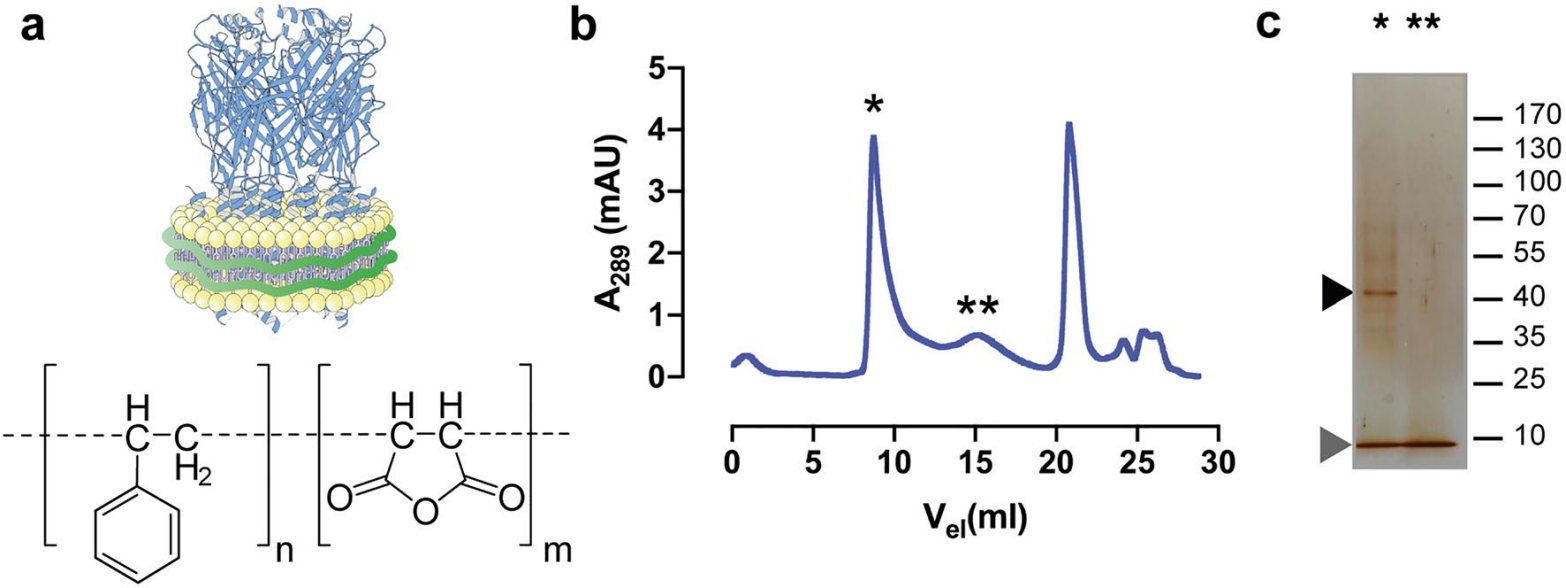
Purification and functional characterization of the α1-His GlyR in SMA-copolymer nanodiscs. (**a**) Schematic representation of GlyR (PDB: 3JAE) in native nanodiscs (upper) and chemical structure of SMA-copolymer (lower) with a ratio of n:m of 2:1 used in this study. Size exclusion chromatogram (**b**) and SDS-PAGE analysis (**c**) showing an efficient separation of α1-His GlyR nanodiscs. Peak fraction (*) shows a clear band (black arrow) between 40 and 55 kDa, corresponding to the α1 GlyR (MW: 48 kDa) and a band migrating at ~ 10 kDa corresponding to SMA copolymer. Gel image was cropped, indicated by a grey cropping line. Subfigure (**a**) was created using Abobe Illustrator CC version 24.3 (https://www.adobe.com/kr/products/illustrator.html).

To probe the general potential of SMA-solubilized GlyRs for pharmacological analysis we solubilized a His-tag fused α1 construct (α1-His) by incubating SMA copolymers with the isolated membrane fraction of α1-His overexpressing HEK293 cells (Fig. 1a). The α1-His containing nanodiscs (Fig. 1a) were further isolated with Ni-affinity chromatography, followed by size-exclusion chromatography (SEC). SDS-PAGE analysis of peak fraction one (Fig. 1b) shows a clear band, migrating at ~ 48 kDa, corresponding to the molecular weight of the α1 GlyR monomer (Fig. 1, Supplementary Fig. S1). The SMA copolymer runs at an expected lower molecular weight^26^ of approximately 10 kDa. The second SEC peak (Fig. 1b) reveals only SMA copolymer without any additional protein band, which represent probably free polymer that was interacting with the Ni-affinity columns. The molecular weight and size of the purified α1 GlyR nanodiscs were further investigated by the generated SEC data. Thus, the nanodiscs having an average molecular weight of 495 kDa with a calculated stoke radius of 13.2 nm, which is in good agreement with previous studies^26–28^. Since the pentameric receptor has a molecular weight of 240 kDa and no accessory proteins in the purified fraction can be detected, we conclude that the residual molecular weight of the nanodiscs must result from its lipid bilayer. We therefore showed that the SMA copolymer solubilization of α1 GlyR in nanodiscs was successful and suitable for further investigation.

### Functional characterization of α1 GlyR nanodiscs by microscale thermophoresis

**Figure 2:**
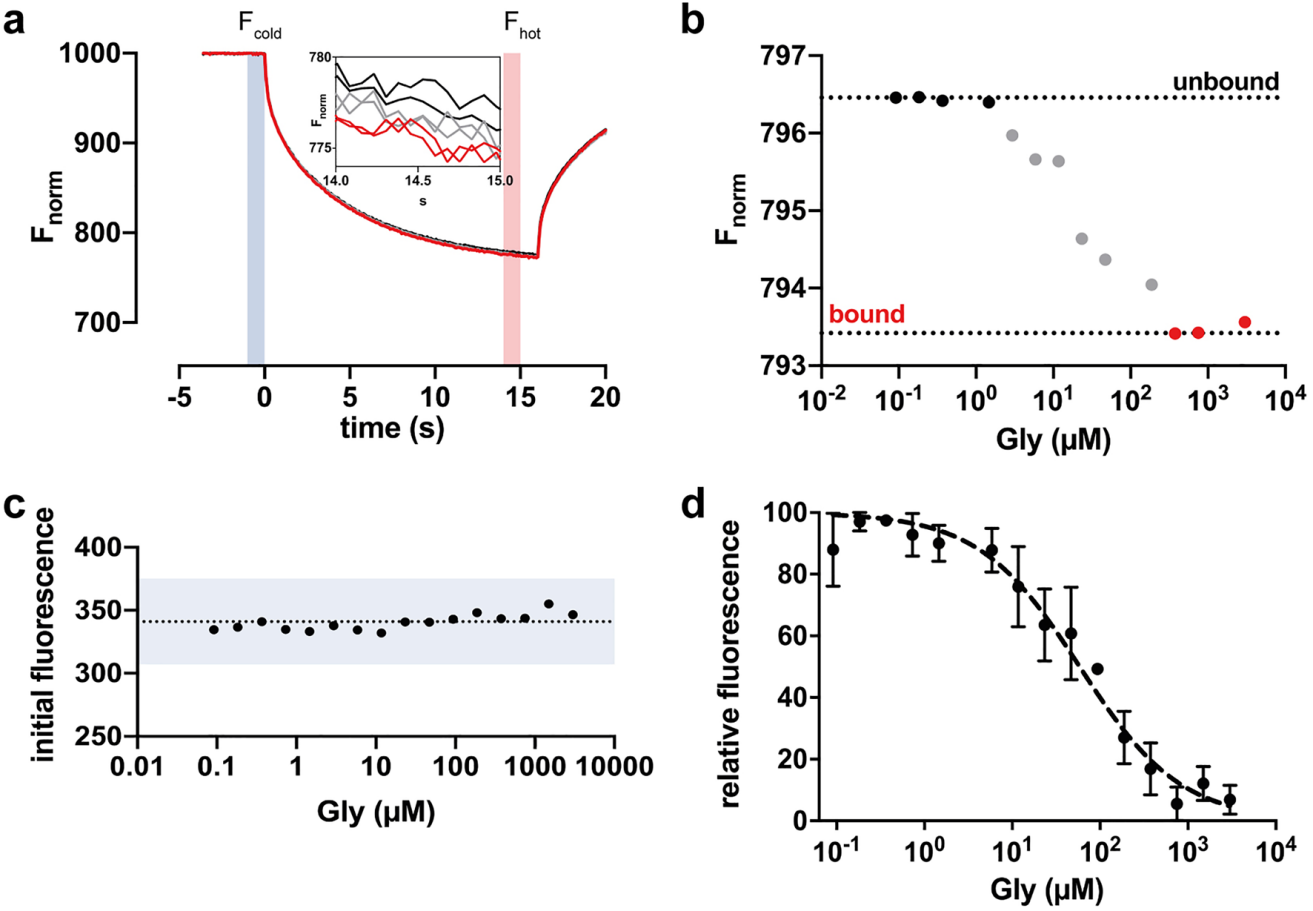
Functional characterization of SMA copolymer solubilized α1-His GlyR. (**a**) Example trace of primary thermophoresis data. Thermophoretic movement of α1-His GlyR nanodiscs is expressed as the change in fluorescence signal between initial fluorescence F_cold_ (0 s) and fluorescence after thermodiffusion F_hot_ (15 s) and was calculated as ratio of both values as described in the “Materials and Methods” section. Inset shows an amplification of representative fluorescence traces of the thermophoretic movement of the fluorescence labeled α1-His GlyR between 14 and 15 s (F_hot_) obtained at different glycine concentrations [0 and 1 (black), 10 and 100 (gray) and 1000 and 3000 µM glycine (red)]. (**b**) The change in thermophoretic movement upon binding of increasing concentrations of Gly results in a change of the relative fluorescence between the unbound state (black) and glycine-bound state (red) after 15 s. (**c**) Initial fluorescence count distribution for each concentration is under 10% and showing no ligand-dependent fluorescence quenching. (**d**) Dose–response curve obtained from MST experiments of α1-His GlyR. Binding of glycine to fluorescence-labeled α1-GlyR was obtained with a titration series from 3 mM to 0.73 µM in PBS buffer, pH 7.4. The change in thermophoretic signal leads to a cEC_50_ of 65 ± 22.8 µM. Error bars represent SEM between *n* = 3 independent experiments.

To examine the functionality of the purified α1 GlyR nanodiscs, we intended to analyze concentration-dependent conformational changes upon binding of the agonist glycine by microscale thermophoresis (MST). Therefore, we diluted the fluorescence labeled α1-His GlyR containing nanodiscs (see “Materials and Methods” section) to a final concentration of 400 nM and added a glycine dilution series of 3 mM down to 0.73 µM. Samples were loaded into glass capillaries and get focused by an infrared (IR) laser, creating a spatial temperature gradient. Thermophoretic movement of the fluorescence labeled α1-His GlyR nanodiscs was measured by fluorescence emission coupled into the IR laser path^29^. We found that addition of glycine to the receptor complex was sufficient to induce a change in thermophoretic mobility of the complex (Fig. 2a). Increasing glycine concentrations resulted in a concentration-dependent shift of the thermophoretic signal saturating at 500 µM glycine (Fig. 2b). Because no concentration-dependent fluorophore quenching could be observed (Fig. 2c), we concluded that the purified SMA copolymer nanodiscs must contain functional pentameric α1 GlyR receptors and that the alterations in particle movement obtained must be due to a glycine-induced alteration of the conformation, size, charge and/or hydration shell^30^ of the GlyR. This is consistent with the findings at native GlyRs where (i) pentameric assembly of the GlyR is required for the proper formation of the specific glycine-binding site at the interface of adjacent subunits and (ii) binding of glycine induces a significant conformational rearrangement of the ECD. Analyzing the glycine depended shift of the thermophoretic movements revealed an EC_50_ value of 65 ± 22.8 µM (mean ± SEM; *n* = 4; Fig. 2d), which is similar to the apparent EC_50_ value obtained by electrophysiology in α1 GlyR expressing HEK293 cells^12,13^. To distinguish electrophysiological and MST-determined EC_50_ values, we termed them in the following as aEC_50_ (a for apparent; e-phys) and cEC_50_ (c for conformation, MST), respectively. In conclusion, our results obtained upon analyzing glycine concentration-dependent conformational changes of the GlyR by MST embedded in SMA copolymer nanodiscs, indicate that the glycine-induced contraction within the ECD leading to a highly efficient gating of the channel pore, resulting in a 1:1 ratio of cEC_50_ and aEC_50_ values.

### Characterization of glycine binding to α1 GlyR obtained from HEK293 cells and *X. laevis* oocytes

**Figure 3:**
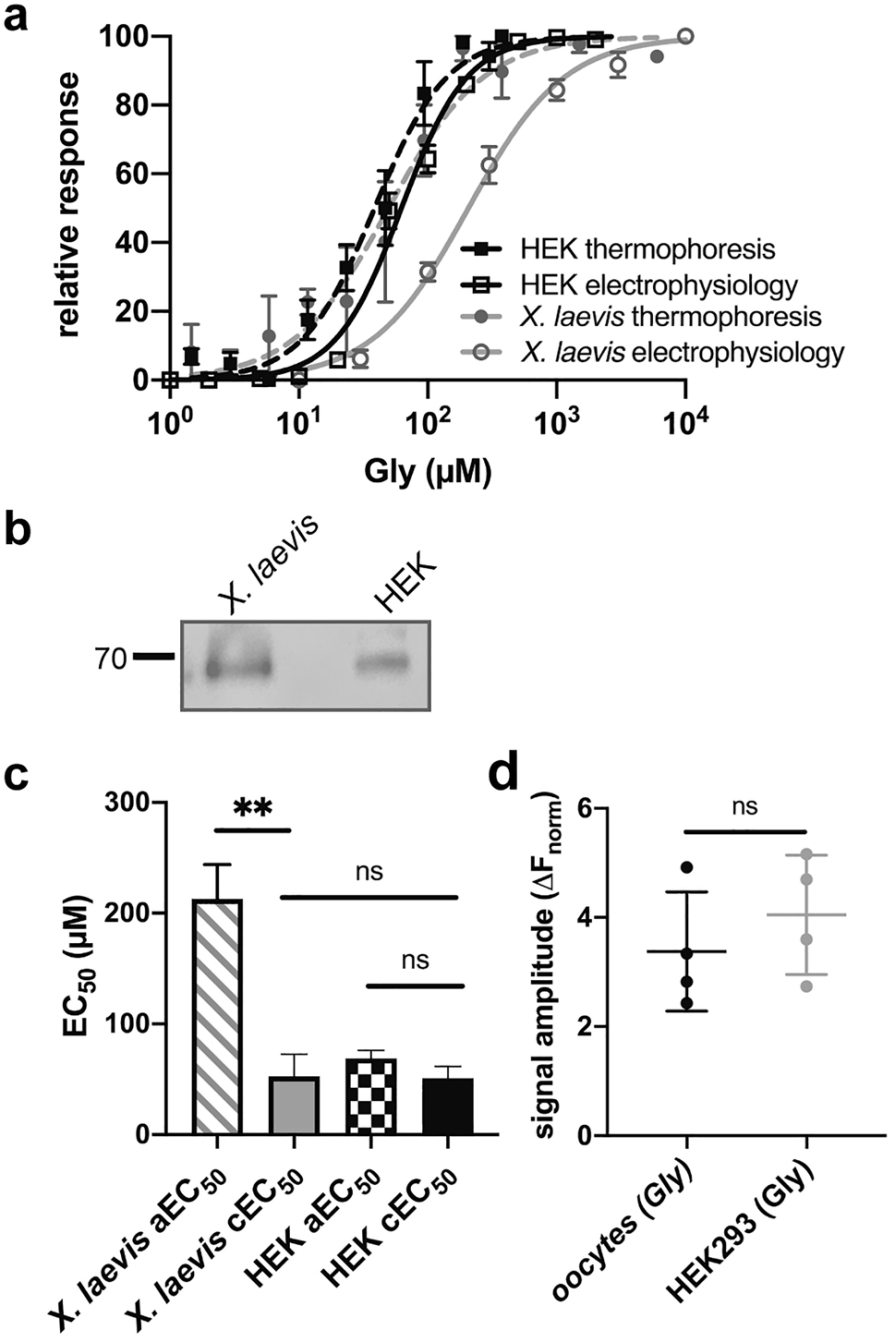
Functional analysis of α1-GFP GlyR nanodiscs from HEK293 cells and *X. laevis* oocytes. (**a**) Dose–response relationship of α1-GFP GlyR obtained by MST experiments and electrophysiological recordings from HEK293 cells and oocytes. MST data points were inversely normalized for better comparison with dose–response curves obtained from electrophysiological measurements. Data are shown in mean ± SEM. (**b**) Western blot of SMA-copolymer solubilized GFP-GlyR α1 obtained from the membrane fractions of oocytes and HEK293 cells, show a single band at the calculated molecular weight below 70 kDa. Western blot image was cropped, indicated by a grey cropping line. (**c**) Electrophysiological experiments obtained from oocytes and HEK293 cells revealed aEC_50_ values 212.9 ± 21 µM and 68.8 ± 7.4 µM, respectively. cEC_50_ values of 52.6 ± 40.8 µM (*n* = 4) and 40.9 ± 13.4 µM (*n* = 4) obtained from oocytes and HEK293 cells showing no significant difference (*p* = 0.41). The aEC_50_ obtained from is significantly higher (*p* < 0.01) than the measured cEC_50_, while the aEC_50_ and cEC_50_ obtained from HEK293 cells show no difference (*p* = 0.23). Error bars represent SEM between independent experiments. (**d**) Signal amplitudes obtained from MST experiments reveal no differences between HEK293 cells (signal amplitude = 4.04 ± 1.09) and oocytes (signal amplitude = 3.38 ± 1.09; *p* = 0.41, *n* = 4). Data are shown in mean ± SD. Unpaired two-side *t* test for statistics.

One key advantage of solubilizing membrane proteins in SMA copolymer nanodiscs is the retention of their native lipid surrounding, enabling the analysis of ligand-induced conformational changes while preserving the influence of the native membrane environment in in vivo-like conditions. Homomeric α1 GlyR exhibit a major discrepancy concerning their glycine affinity with aEC_50_ values of 212.9 ± 21 µM and 68.8 ± 7.4 µM when heterologously expressed in *X. laevis* oocytes or HEK293 cells, respectively (Fig. 3a). The root cause and underlying mechanism of these variations in the efficiency of receptor activation across different expression systems is still unknown. To further explore this concept we analyzed the binding affinity for α1 GlyR extracted from HEK293 cells and oocytes. To maximally mimic the complex environmental conditions found in the cells and to simplify the analytical process, we used an N-terminal GFP fused receptor (α1-GFP GlyR) construct, that was directly measured after SMA-solubilization within the total nanodiscs fraction, that has previously reported for soluble GFP fused proteins^29^. Western blot analysis (Fig. 3b, Supplementary Fig. S2) of the solubilized membrane fractions indicates an adequate incorporation of GlyRs into SMA copolymer nanodiscs. For comparable results, we first probed the binding affinity for glycine from HEK293 cell lysate. α1-GFP GlyRs also showed a concentration-dependent shift in their thermophoretic mobility, similar to purified and fluorescence labeled His-tagged α1 GlyR, with a cEC_50_ value of 40.9 ± 13.4 µM (*p* = 0.22, *n* = 4). A concentration-dependent GFP quenching was not detected as well (not shown). Thus our GFP tagged α1 GlyR yield similar cEC_50_ values compared to pure α1 GlyR nanodiscs and was suitable for the comparative analysis of α1 GlyRs expressed in oocytes. Next we analyzed the apparent glycine binding affinities of α1 GlyR extracted from oocyte membranes (Fig. 3a,c). The determined cEC_50_ of 52.6 ± 40.8 µM (*n* = 4) for glycine reveals no significant difference (*p* = 0.58; Fig. 3c) compared with the cEC_50_ value obtained from HEK293 cells. Thus, the cEC_50_ value obtained from HEK293 cells is identical with the aEC_50_ (*p* = 0.23), whereas the cEC_50_ value obtained from oocytes is about 4 times lower than the aEC_50_ (*p* < 0.01). Furthermore, the similar thermophoretic signal amplitudes (*p* = 0.41; Fig. 3d) obtained from both expression systems indicate that the GlyR adopts the same ECD configuration with a similar degree of domain-closure upon glycine binding. These findings implicate, that the efficiency of glycine to induce the conformational change within the ECD is identical in both expression systems and the increased aEC_50_ observed in oocytes must be linked to an impaired channel opening.

### Conformational changes induced by binding of the partial agonist taurine

**Figure 4:**
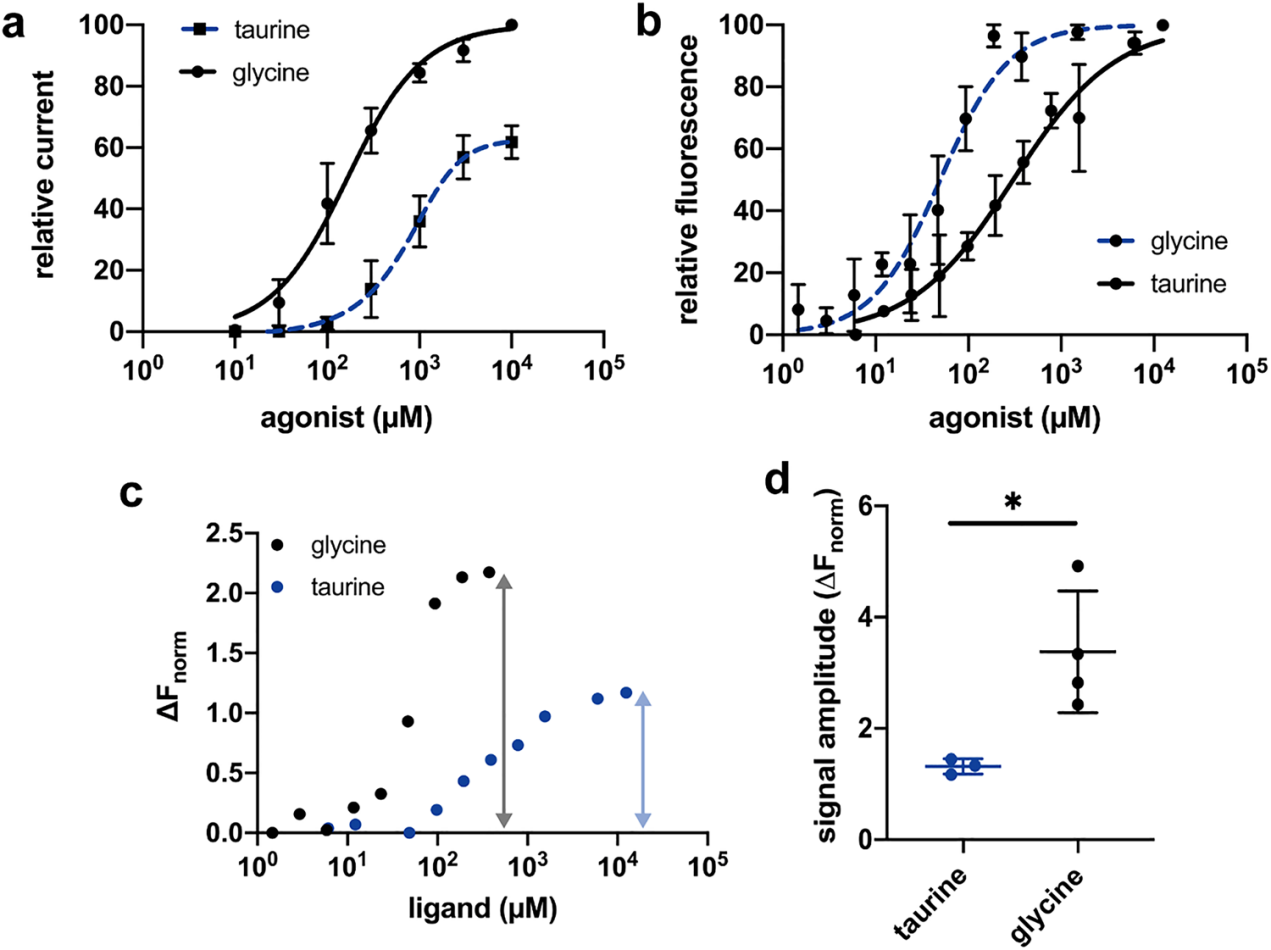
Binding characteristics of the partial agonist taurine to α1-GFP GlyR nanodiscs. (**a**) Dose–response data for glycine and taurine of heterologous expressed α1 GlyR from *X. laevis* oocytes. Taurine acts as a partial agonist with an aEC_50_ value of 843 ± 16 µM reaching a maximum current of 61% compared to glycine (*n* = 3). Taurine currents are normalized to the maximum glycine currents for each cell. Dose–response data of glycine are the same as shown in Fig. 3. Error bars represent SEM. (**b**) MST binding experiment of α1-GFP GlyR with a taurine titration series of 6 µM to 12.5 mM results in a cEC_50_ value of 473.8 ± 46.1 µM (*n* = 3). Error bars represent SEM. (**c**) Exemplary α1-GFP GlyR MST data of taurine (blue circles) and glycine (black circles) obtained from oocytes displaying a difference in their maximal thermophoretic mobility (grey and blue arrows). (**d**) Comparison of the signal amplitudes of α1-GFP GlyR SMALPs expressed in HEK293 cells and oocytes for glycine and taurine. Binding of taurine leads to a significant decreased thermophoretic movement (*p* = 0.024, unpaired two-side *t* test, *n* = 3) with signal amplitudes of 1.32 ± 0.14 compared to glycine-bound receptors with signal amplitudes of 3.38 ± 1.09. Data are shown in mean ± SD.

Taurine acts as a partial agonist on α1 GlyR in oocytes with an aEC_50_ value of 843 ± 16 µM (*n* = 3) and a maximal current of 61% compared to glycine (Fig. 4a). To investigate how taurine influences the ECD conformation and if glycine may act as a partial agonist in oocytes, we analyzed the conformational transformation of α1-GFP GlyR containing SMA nanodiscs in response to taurine by MST, as well. As expected, the cEC_50_ value of 473.8 ± 66.1 µM (*n* = 3; Fig. 4b) is much higher than for glycine and is approximately half as much as the obtained aEC_50_ value (*n* = 3; *p* < 0.05). Also the Hill coefficient of 0.8 decreased for taurine, which is in good agreement with previous studies, reporting a decrease for less efficient agonists^15,31^. Since the binding of the partial agonist taurine might stabilize a conformational state with an altered ECD closure, we analyzed the discrepancies in thermophoretic movement between the unbound and agonist bound states. We found, that the signal amplitude between glycine and taurine significantly decreases (*p* < 0.05, Fig. 4c,d) from 3.38 ± 1.09 to 1.32 ± 0.17, respectively. This finding implicates, that taurine stabilizes a distinct conformational state under saturating conditions that can be clearly distinguished from the conformational state adopted after glycine binding. In contrast, binding of glycine to the GlyR induces in both expression systems the same overall conformational configuration, leading to the conclusion that glycine acts not as a partial agonist in oocytes like taurine.

## Discussion

In this study, we characterized the conformational states of the homomeric α1 GlyR in SMA copolymer nanodiscs by MST during receptor activation by glycine and the partial agonist taurine upon heterologous expression in HEK293 cells and *Xenopus* oocytes. Our results indicate that in the GlyR (i) partial and full agonists induce different conformational transitions and (ii) conformational transitions after agonist-binding affect apparent affinity. Thus our data shed light on the conformational features and mechanisms determining receptor activation by different agonists in different cell systems in the pLGIC superfamily.

In principle, two different models for the conformational states induced by a full and partial agonist at pLGICs can be envisaged. First, binding of the agonist simply shifts the equilibrium between the closed state and the open state of the channel. This model is based on the initial work by del Castillo and Katz^32^, where it has been supposed that full and partial agonists differ in their efficiency to activate a receptor, implying that both partial and full agonists stabilize the same open conformational state of the receptor. Consequently, a full agonist would promote efficient channel opening, while a partial agonist would be less effective in stabilizing the open state. Second, it can be assumed that a full and a partial agonist can adopt different conformational states in the agonist binding domain which are correlated to the extent of channel opening, mainly depending on the specific stereochemical properties of the ligand. Recent studies at the GlyR indicate that receptor activation includes one or more intermediate states, so called flipped or primed states, where the ECD is closed but the channel is still shut^9,10,20^. In this model, the origin of partial agonism would be related to a reduced ability of the receptor to reach an intermediated pre-open state, rather than a reduced ability to fully activate the receptor, when the intermediated is reached^10,33^. Nevertheless, little is known if the altered receptor kinetic for partial agonists is also reflected by distinct conformational states, as seen in ionotropic glutamate receptors^34,35^.

**Figure 5:**
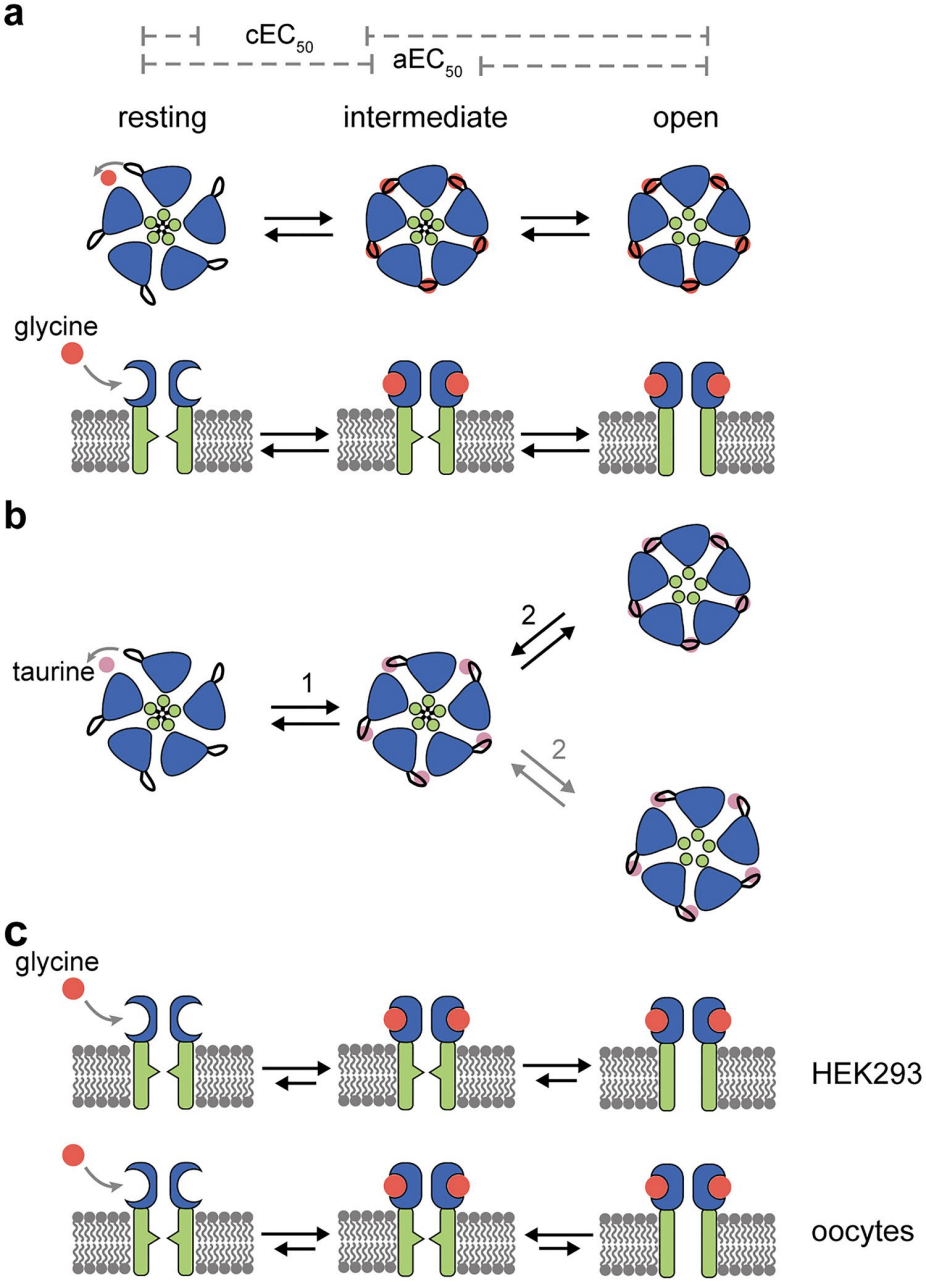
Schematic model of the activation mechanism of GlyRs for full and partial agonists. (**a**) The general activation mechanism of GlyRs includes at least three conformational states, whereby two conformations adopting a contracted ligand-bound ECD. Binding of a full agonist (red dot) induced an ECD closure of the resting receptor, while the ion channel is still shut (intermediate state). This ECD closure finally opens the ion channel and activates the receptor (open state). The ability of an agonist to change the conformation within the ECD (cEC_50_) was measured by MST, while the general ability to activate the receptor (aEC_50_) was determined by electrophysiological methods. (**b**) Binding of a partial agonist (purple) initiate an incomplete closure of the ECD (1). The receptor is either activated by a further ECD closure (2, grey) that leads to an ion channel opening (open state) or can directly open with a less contracted ECD (2, black). (**c**) GlyR activation in HEK293 cells and oocytes. Glycine binding to GlyRs in HEK293 cells is characterized by a contraction of the ECD and a rapid channel opening, reflected by similar cEC_50_ and aEC_50_ values. Binding of glycine to GlyRs in oocytes is also characterized by an efficient reorientation of the ECD with an impaired channel opening, possibly stabilizing an intermediate state with a contracted ECD and a closed channel pore. Figure was drawn using Abobe Illustrator CC version 24.3 (https://www.adobe.com/kr/products/illustrator.html).

Our thermophoretic analysis during receptor activation induced by agonists reveals large conformational changes of the GlyR. We attribute the detected agonist-induced rearrangements to a contraction within the ECD entrapping the ligand between adjacent subunits^8,36,37^, rather than structural changes within the channel pore itself, which are masked by the surrounding lipid-bilayer of the nanodisc. This is consistent with a recent finding that, although full and partial agonists of the GlyR have the same orientation within the ECD upon binding, interactions between the amino groups of the agonists and receptor residues differ remarkably resulting in a different extent of ECD contraction with the volume of the agonist binding pocket smallest in the glycine-bound structure^38^. Since our measurements were performed under equilibrium conditions, our thermophoretic data therefore most likely reflect structural changes between the resting conformation, with an open ECD, and one or multiple intermediate states, as well as the open state, were the ECD is contracted (Fig. 5a). Thus, our MST measurements would reflect exclusively the agonist-induced conformational change within the ECD, whereas the electrophysiological measurements determine the general ability of the agonist to open the ion channel (aEC_50_ value). Most strikingly, our MST measurements revealed different conformational changes of the ECD induced by taurine compared to the full agonist glycine. Therefore, we assume that the interactions between taurine and the ECD loops result in a lesser extend of ECD contraction, and therefore to the observed decreased thermophoretic mobility. This indicates that glycine- and taurine-binding induces a different degree of conformational change in the ECD and that the extent of agonist-induced ECD closure is correlated with the efficacy of agonists.

Since we cannot discriminate in our MST measurements between distinct closed ECD conformations, two general activation models for partial agonists can be envisaged (Fig. 5b). First, the taurine-induced conformation reflects a closed intermediate state that further adopts the same closed ECD conformation as seen for glycine during full activation (Fig. 5b, upper row). Second, the taurine-induced conformation reflects the final open conformation, without reaching a fully contracted ECD conformation (Fig. 5b, lower row). However, recent findings support the existence of an intermediated taurine-bound state, that is characterized by a lesser degree of ECD rotation^38^.

Depending on the cell system analyzed, the GlyR displays in electrophysiological measurements a remarkable difference in the apparent affinity (aEC_50_) of its agonists^4^. Thus, the apparent affinity of an agonist is not exclusively determined by its specific stereochemical properties. Previous studies supposed that intermolecular cooperativity at higher receptor expression levels may influence agonist affinity in different cell systems^39^. Our experiments show that the large variability of the aEC_50_ for glycine observed in different cell systems (HEK293 vs. oocyte) is not correlated with the efficiency to induce a contraction within the ECD (cEC_50_) after glycine binding. In addition, the cEC_50_ values in HEK293 and oocytes are similar to the aEC_50_ value obtained from electrophysiological measurements in HEK293 cells, indicating an efficient receptor activation in HEK293 cells once the ECD has closed, consistent with previous single-channel measurements^9,10,20^. As a direct consequence, the receptor equilibrium lies strongly on the ligand-bound, open conformation. In contrast, while the cEC_50_ is unchanged in oocytes, the aEC_50_ apparent affinity is decreased. We therefore conclude, that the decreased apparent affinity of GlyR in oocytes probably arise from a limited ability to open the channel pore once the ECD is closed, rather than the ability to induce a conformational change in the ECD layer upon glycine binding (Fig. 5c). We speculate that the impaired coupling of the ECD and the TMD is driven (i) by lipid or sterol modulations, as described for other members of pLGICs^40–42^ and supported by recent findings supposing interactions between glycine receptors and cholesterol^43^ or (ii) by the impact of the ICD^44^. This would explain the high variability of aEC_50_ values seen upon electrophysiological measurements of GlyRs in the brain and in different heterologous expression systems. Thus, the apparent agonist-affinities of the GlyR obtained in electrophysiological measurements are likely determined by conformational transitions after the agonist-induced ECD closure which is in agreement with several postulated intermediate shut states between the resting and open conformation, as indicated by different primed or flipped schemas. Our data may also have some implications for the understanding of the differential impact of (i) mutations^45^ and modulators^25^ in the ECD and (ii) the TMD and ICD^44^ at the GlyR in affecting partial agonism. Therefore mutations and modulators acting in the ECD might affect the conformational change in the ECD layer upon agonist binding whereas modulators acting in the TMD and the ICD may alter intermediate shut states between the resting and open conformation. Future work will need to explore these possibilities by testing the effects of mutations, modulators and the ICD on conformational states by thermophoretic analysis of the GlyR extracted in SMA-copolymer nanodiscs enabling the receptor to occupy physiologically relevant states.

In summary, by analyzing conformational states of the GlyR by MST, our results indicate that first partial agonism in the pLGIC family is reflected by the adaption of distinct receptor conformations and second that modulation within the TMD region and/or the ICD causes the variable apparent affinities seen in different cell systems. Therefore, our approach provides an easy access to correlate structural and functional impacts of ligand binding and may also help in the mechanistic understanding of positive allosteric modulators as well as in rationalized drug design.

## Supplementary information

Supplementary Figures.

## Data Availability

All data supporting the findings of this study are available within the article or are available from the corresponding author upon reasonable request. All cDNA constructs are available from the corresponding author based on reasonable request.
