# Supplementary material for: Thermophoretic analysis of ligand-specific conformational states of the inhibitory glycine receptor embedded in copolymer nanodiscs
Source: Sci Rep. 2020 Oct 6;10:16569. doi: 10.1038/s41598-020-73157-2 (PMC7538598; doi:10.1038/s41598-020-73157-2)
Supplement: Supplementary file 1 — Supplementary Figures. [file 41598_2020_73157_MOESM1_ESM.pdf]

Thermophoretic analysis of ligand-specific conformational states of the inhibitory glycine receptor embedded in copolymer nanodiscs

Max Bernhard[1], Bodo Laube[1,2]

[1]Department of Biology, Neurophysiology and Neurosensory Systems, Technical Universityof Darmstadt, Schnittspahnstrasse 3, 64287 Darmstadt, Germany
[2]Centre for Synthetic Biology, Technical University of Darmstadt, 64283 Darmstadt, Germany

Corresponding author: laube@bio.tu-darmstadt.de; Tel.: +49-6151-16-20970

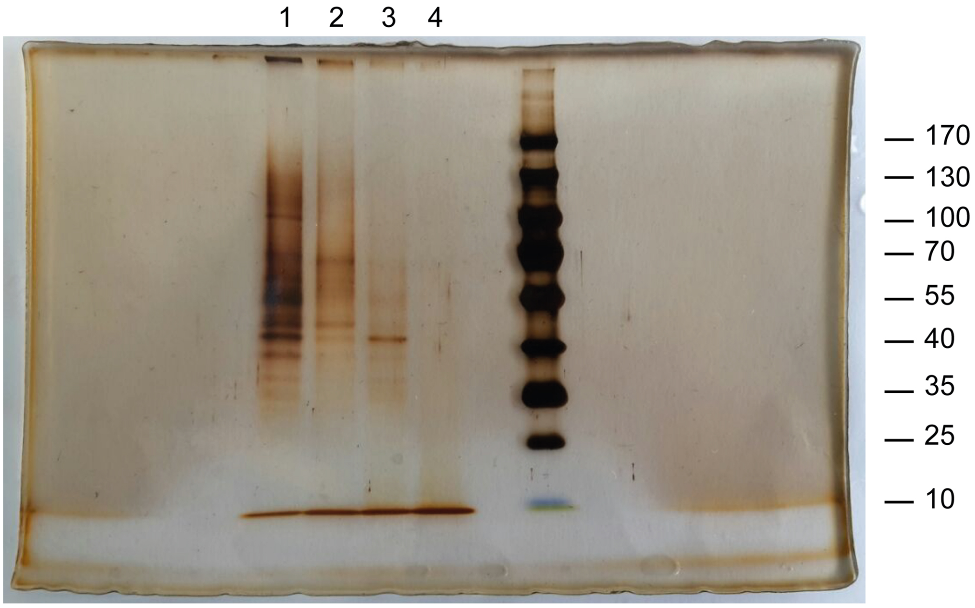

Supplementary Figure S1: Full size SDS-PAGE gel image after Ni-NTA purification and size exclusion chromatography as of α1-His GlyR nanodiscs shown in Fig. 1c. Total Flowthrough (lane 1) and pooled elution fractions (lane 2) after Ni-NTA purification. Lane 3 and 4 showing the peak fractions * and ** of Size exclusion chromatogram in Fig. 1b. Peak fraction (*) shows a clear band (black arrow) between 40 kDa and 55 kDa, corresponding to the α1 GlyR (MW: 48 kDa) and a band migrating at ~10 kDa corresponding to SMA copolymer.

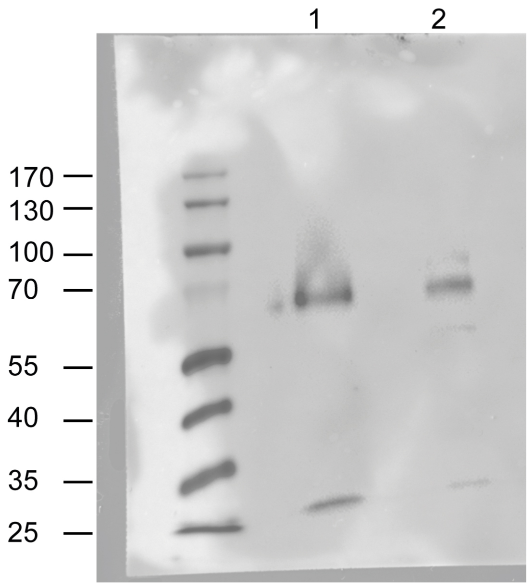

Supplementary Figure S2: Uncropped Western blot gel image of Fig. 2b. SMA-copolymer solubilized GFP-GlyR α1 obtained from the membrane fractions of oocytes (1) and HEK293 cells (2), show a single band at the calculated molecular weight below 70 kDa.
